# Stem cell biology is population biology: differentiation of hematopoietic multipotent progenitors to common lymphoid and myeloid progenitors

**DOI:** 10.1186/1742-4682-10-5

**Published:** 2013-01-17

**Authors:** Marc Mangel, Michael B Bonsall

**Affiliations:** 1 Department of Applied Mathematics and Statistics, University of California, Santa Cruz, CA, USA; 2 Department of Biology, University of Bergen, Bergen, Norway; 3 Mathematical Ecology Research Group, Department of Zoology, University of Oxford and St Peter’s College, Oxford, UK

**Keywords:** Hematopoieitic stem cell, Multipotent progenitor, Common lymphoid progenitor, Common myeloid progenitor, Darwinian fitness, Natural selection, Population dynamics

## Abstract

The hematopoietic stem cell (HSC) system is a demand control system, with the demand coming from the organism, since the products of the common myeloid and lymphoid progenitor (CMP, CLP respectively) cells are essential for activity and defense against disease. We show how ideas from population biology (combining population dynamics and evolutionary considerations) can illuminate the feedback control of the HSC system by the fully differentiated products, which has recently been verified experimentally. We develop models for the penultimate differentiation of HSC Multipotent Progenitors (MPPs) into CLP and CMP and introduce two concepts from population biology into stem cell biology. The first concept is the Multipotent Progenitor Commitment Response (MPCR) which is the probability that a multipotent progenitor cell follows a CLP route rather than a CMP route. The second concept is the link between the MPCR and a measure of Darwinian fitness associated with organismal performance and the levels of differentiated lymphoid and myeloid cells. We show that many MPCRs are consistent with homeostasis, but that they will lead to different dynamics of cells and signals following a wound or injury and thus have different consequences for Darwinian fitness. We show how coupling considerations of life history to dynamics of the HSC system and its products allows one to compute the selective pressures on cellular processes. We discuss ways that this framework can be used and extended.

## Introduction

Hematopoiesis (the formation of blood components) is a highly orchestrated and dynamical process. Hematopoietic Stem Cells (HSCs) give rise, through a large array of successively differentiated progeny, to mature blood cells. While progress has been made in understanding the HSC system, particularly at the molecular level [1,2], Tan et al. (pg 82-83) [3] concluded a recent review on HSCs by identifying critical unanswered questions: 1) Is it possible to manipulate adult stem cells to increase their ability to proliferate in vitro while maintaining stem cell qualities, so that adult stem cells can be used as a sufficient source of tissue for transplants and other therapeutic strategies? 2) What are the intrinsic and extrinsic controls that keep stem cells from differentiating or that direct them along a particular differentiation pathway to form one specialized cell type rather than another? 3) What are the factors responsible for stem cell responses to injury or damage that enable rapid activity and appropriate contribution to tissue repair and regeneration? 4) Can the ‘stress’ signals’ that command facultative stem cells to respond to tissue damage and gain specific regenerative quality be harnessed for therapeutic value?

We will show that these questions can only be fully answered if one considers the connection between the needs of the organism and the HSC system, a demand control system [4] in which the demand comes from the organism via signals to the HSC system. As noted by Metcalf [4] in his classic lectures, appropriate long-term behavior of the HSC system is essential for the health of the organism. Furthermore, HSCs are required to produce the required differentiated cells without depleting the stem cell pool or creating damaged stem cells that lead to cancer. Even in the absence of oxidative damage due to ischemia/reperfusion, blood loss, or infections damage to blood cells and tissues caused by reactive oxygen species is a common process leading to cell apoptosis, and mature blood cells have a steady rate of turnover and are thus constantly replaced [5].

Thus, organismal performance is intimately connected to HSCs and their products [6-9]. The myeloid products of the HSC system are particularly important for antipredator and foraging behaviors and general immune response and the lymphoid products of the HSC system are essential for specific immune response and neonatal survival. Recent whole organism manipulations have demonstrated the veracity of this proposition (see [10] for myeloid cells; [11] for lymphoid cells). At the same time, when the organism is in a steady state (homeostasis) the HSC system and its products are relatively stable [12]. In addition, the demand is not unlimited; for example, it has been known for a very long time that organismal performance is a peaked function of hematocrit (e.g., [6,9,13]).

**Figure 1:**
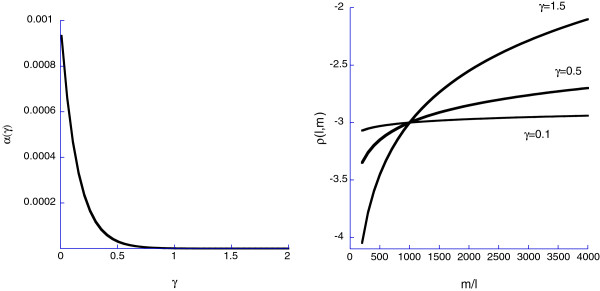
**A diagrammatic derivation of Eqns** 1 **to** 6 **(details given in Additional file**1**). a)** In the most general case, we consider stem cells (S), a series of Multipotent Progenitor Cells (MPP), a Common Lymphoid Progenitor (CLP) and a Common Myeloid Progenitor (CMP). CLPs give rise to B, NK, and T cells; CMPs give rise to Erythrocytes (E), Granulocytes (G), and Platelets (P). We denote the total numbers of lymphoid and myeloid cells by L and M respectively, rates of differentiation by *r*_·_(with subscript indicating the cell type involved), rates of development of MPP cells by *λ*_·_, feedback from fully differentiated cells on those rates by *Φ*_·_, and rates of cell death by *μ*_·_. The feedback functions have the property that they are 1 when stem cell or fully differentiated cell numbers are low and decline as stem cells or fully differentiated cells increase. Thus, for example, stem cells renew (one stem cell becomes two) at rate *r*_*s*_*Φ*_*s*_(*l*,*m*)when the concentrations of lymphoid and myeloid cells are *l* and *m* respectively, asymmetrically differentiate (one stem cell becomes two stage-0 progenitors) at rate $2r_{p^{\prime }}\Phi_{p^{\prime }}(l,m)\Phi_{s}(l,m)$, symmetrically differentiate (one stem cell becomes a stem cell and a stage-0 progenitor) at rate *r*_*p*_*Φ*_*s*_(*l*,*m*), and die at rate *μ*_*s*_. Similar interpretations hold for other transitions. The Multipotent Commitment Response (MPCR), denoted by *ρ*(*l*,*m*), is the probability that a MPP in its final stage commits to the lymphoid route. **b)** To focus on the MPCR, we combine all of the fully differentiated cells into lymphoid and myeloid classes (L and M) and use Michaelis-Menten-like arguments to compress the MPP class into a single stage, assuming that steady states of intermediate stages are rapidly reached, characterized by combination of rate constants *Ω*_*N*_.

In this paper, we show that the questions raised by Tan et al. [3] are answered most effectively if one takes an approach to stem cell biology based on population biology, which involves combining the dynamics of cells with considerations of fitness and natural selection [14]. We develop a theoretical framework that complements existing models (Additional file 1: Table S1) to explore the dynamics of the HSC system. Elsewhere ([15]; Figure 1 here) we have developed stochastic and deterministic models of HSC cells and their associated products and applied evolutionary invasion analysis [16] and state dependent life history theory [17-20] to show that understanding the dynamics of HSCs and their products requires asking more than whether a stem cell renews, symmetrically differentiates, or asymmetrically differentiates. Understanding the roles of positive and negative feedback is essential for predicting stem cell dynamics. By linking these feedback processes to stochastic population models (which allow uncertainties inherent in the system to be accounted for) we showed how well the overall mean dynamics of the system can be approximated a system of ordinary differential equations. We build on this work here and introduce additional concepts associated with population biology to the biology of stem cells, with the focus on the HSC system. We introduce the *Multipotent Progenitor Commitment Response (MPCR)* that characterizes the penultimate differentiation of a multipotent progenitor (MPP) to a Common Lymphoid Progenitor (CLP) or a Common Myeloid Progenitor (CMP), i.e. whether they follow a myeloid or lymphoid track. Although we recognize that within the myeloid track there is another decision towards a granulocyte-macrophage progenitor or megakaryocyte-erythrocyte progenitor (see Additional file 1). We show how the fitness (survival and reproduction) of the organism shapes the MPCR, thus providing an approach for modeling the demand control nature of the HSC system.

## Methods

We begin first by describing, in summary here, with details in Additional file 1, the dynamics of the stem cells and their descendants, after which we describe the components of fitness (survival and the reproduction) and their dynamics. We then couple the two together.

### Dynamics of stem cells and their descendants

In the Additional file 1, we derive the computational model given below and in Figure 1 we provide a graphical representation of the full mathematical model. We thus consider stem cells, with concentration denoted by [*S*], Multipotent Progenitor cells, denoted by [*MPP*], Common Lymphoid Progenitors, denoted by [*CLP*], Common Myeloid Progenitors, denoted by [*CMP*], fully differentiated lymphoid cells, denoted by [*L*](measured in numbers per milliliter), and fully differentiated myeloid cells, denoted by [*M*](measured in numbers per nanoliter). Central to these dynamics we assume that the stem cell niche can support at most *K* stem cells and that in absence of all other feedback (described below), the dynamics in the niche follow Gompertzian kinetics (justified in [15]).

The dynamics are described by the following set of coupled ordinary differential equation:

(1) $$\begin{array}{lll} & \;\frac{d[S]}{\text{dt}}=\;[S]\cdot \text{log}(K/[S])(r_{s}-r_{p^{\prime }}\Phi_{p^{\prime }}([L],[M]))\; & \;\\ & \;\;\;\times \Phi_{s}([L],[M])-\mu_{s}[S]\; \end{array}$$

(2) $$\begin{array}{ll} & \;\frac{d[\text{MPP}]}{\text{dt}}=\;[S]\cdot \text{log}(K/[S])(r_{s}+2r_{p^{\prime }}\Phi_{p^{\prime }}([L],[M]))\; \end{array}$$

(3) $$\begin{array}{lll} & \;\;\;\times \Phi_{s}([L],[M])+(\lambda -r_{d,\text{MPP}})\Phi_{p}([L],[M])\; & \;\\ & \;\;\;\;\times [\text{MPP}]-\mu_{p}[\text{MPP}]\; & \;\\ & \;\frac{d[\text{CLP}]}{\text{dt}}=\;r_{d,\text{MPP}}\Phi_{p}(L,M)\rho ([L],[M])\Omega_{N}[\text{MPP}]\; & \;\\ & \;\;\;-r_{\text{CLP}}[\text{CLP}]-\mu_{\text{CLP}}[\text{CLP}]\; \end{array}$$

(4) $$\begin{array}{lll} & \;\frac{d[\text{CMP}]}{\text{dt}}=\;r_{d,\text{MPP}}\Phi_{p}(L,M)(1-\rho ([L],[M)])\Omega_{N}[\text{MPP}]\; & \;\\ & \;\;\;-r_{\text{CMP}}[\text{CMP}]-\mu_{\text{CMP}}[\text{CMP}]\; \end{array}$$

(5) $$\begin{array}{ll} & \;\frac{d[L]}{\text{dt}}=\;r_{\text{CLP}}[\text{CLP}]+(r_{l}-\mu_{l}-\mu_{l*}I_{V(t)>v_{\text{th}}})[L]\; \end{array}$$

(6) $$\begin{array}{ll} & \;\frac{d[M]}{\text{dt}}=\;r_{\text{CMP}}[\text{CMP}]+(r_{m}-\mu_{m})[M].\; \end{array}$$

where *Φ*_*s*_([*L*],[*M*]), $\Phi_{p^{\prime }}([L],[M])$ and *Φ*_*p*_([*L*],[*M*]) are feedback functions (see below) for the activity of stem cells (*s*), the asymmetric differentiation of stem cells (*p*^′^), and the activity of MPP cells (*p*), respectively. *ρ*([*L*],[*M*])is the demand control function (see below) that describes the probability of an MPP cell differentiating into a lymphoid or myeloid progenitor. *Ω*_*N*_[*MPP*] is the survival of MPP cells from initial differentiation through to ultimate differentiation into CLP or CMP cells (see Additional file 1 for its full derivation). *μ*_*l*∗_characterizes the additional mortality when the immune system is activated and *I*_*a*>*b*_ is an indicator function that is 1 if *a*>*b*and 0 otherwise. All other parameters are defined in Table 1.

**Table 1:** Variables, parameters, their interpretation, and values

***Symbol***	***Interpretation***	***Value***
*t*	Non-dimensional time	1-3500
[*S*]	Concentration of stem cells at time *t*	Eqn 1
[*MPP*]	Concentration of Multipotent Progenitor (MPP) cells at time *t*	Eqn 2
[*CLP*]	Concentration of Common Lymphoid Progenitor (CLP) cells at time *t*	Eqn 3
[*CMP*]	Concentration of Common Myeloid Progenitor (CMP) cells at time *t*	Eqn 4
[*L*]	Concentration of fully differentiated Lymphoid (L) cells at time *t*	Eqn 5
[*M*]	Concentration of fully differentiated Myeloid (M) cells at time *t*	Eqn 6
*K*	Maximum number of stem cells in a niche	10
*r*_*s*_	Maximum rate of stem cell self-renewal	2.5
$r_{p^{\prime }}$	Maximum rate of stem cell asymmetrical division	0.001
$\Phi_{p}^{\prime }([L],[M])$	Feedback control from fully differentiated cells to asymmetric division	Eqn 9
*Φ*_*s*_([*L*],[*M*])	Feedback control from fully differentiated cells to stem cell self-reneval	Eqn 7
*Φ*_*p*_([*L*],[*M*])	Feedback control from fully differentiated cells to symmetric division	Eqn 8
*μ*_*s*_	Rate of stem cell death	0.004
*λ*	Rate of MPP multiplication	0.25
*μ*_*p*_	Rate of MPP cell death	0.02
*Ω*_*N*_	Combination of intermediate multipotent progenitor rate constants	1.0
*r*_*CLP*_	Rate of division of CLP into fully differentiated lymphoid cells	0.01
*μ*_*CLP*_	Rate of CLP cell death	0.001
*r*_*l*_	Rate of multiplication of lymphoid cells	0.025
*μ*_*l*_	Rate of lymphoid cell death when immune system is not activated	0.028
*μ*_*l*∗_	Additional rate of lymphoid cell death when immune system is activated	0.01
*I*_*a*>*b*_	Indicator function for the inequality	=1 if *a*>*b*, 0 otherwise
*v*_*th*_	Threshold concentration for pathogens to activate the immune system	0.025
*r*_*CMP*_	Rate of division of CMP into fully differentiated myeloid cells	0.01
*μ*_*CMP*_	Rate of CMP cell death	0.001
*r*_*m*_	Rate of multiplication of myeloid cells	0.0
*μ*_*m*_	Rate of myeloid cell death	0.01
*l*	Value of [L]	varies
*m*	Value of [M]	varies
*ϕ*_*sl*_(*l*)	Feedback control of fully differentiated lymphoid cells on stem cell activity	Eqn 10
*ϕ*_*sm*_(*m*)	Feedback control of fully differentiated myeloid cells on stem cell activity	Similar to Eqn 10
*ϕ*_*pl*_(*l*)	Feedback control of fully differentiated lymphoid cells on symmetric renewal	Eqn 11
*ϕ*_*pm*_(*m*)	Feedback control of fully differentiated myeloid cells on symmetric renewal	Similar to Eqn 10
$\phi_{p^{\prime }l}(l)$	Feedback control of fully differentiated lymphoid cells on asymmetric renewal	Eqn 11
$\phi_{p^{\prime }m}(m)$	Feedback control of fully differentiated myeloid cells on asymmetric renewal	Similar to Eqn 10
*α*_*sl*_	Feedback parameter in *ϕ*_*sl*_(*l*)	10
*α*_*pl*_	Feedback parameter in *ϕ*_*pl*_(*l*)	100
$\alpha_{p^{\prime }l}$	Feedback parameter in $\phi_{p^{\prime }l}(l)$	20
*α*_*sm*_	Feedback parameter in *ϕ*_*sm*_(*m*)	0.1
*α*_*pm*_	Feedback parameter in *ϕ*_*pm*_(*m*)	0.001
$\alpha_{p^{\prime }m}$	Feedback parameter in $\phi_{p^{\prime }m}(m)$	0.2
*α*	Coefficient in MPP Commitment Response (MPCR)	Varies
*γ*	Exponent in MPCR	Varies
*κ*_*l*_	Density of lymphoid cells in homeostasis	30
*κ*_*m*_	Density of myeloid cells in homeostasis	30,000
*ρ*_*h*_	Fraction of lymphoid cells in homeostasis	Eqn 15
*Δf*([*M*]	Rate of accumulation of fitness when myeloid cell concentration is [*M*]	Eqn 17
F(t)	Fitness accumulated to time *t*	Eqn 18
*ε*	Ratio of organismal to cellular time scale	0.05
*S*(*t*)	Survival to time *t*	Eqn 21
*μ*_*e*_([*M*])	Total rate of mortality when myeloid cell concentration is [*M*]	Eqn 20
*μ*_*e*0_	Myeloid independent rate of mortality	0.05
*μ*_*e*1_	Myeloid dependent rate of mortality	5.0
*μ*_*i*_([*V*])	Additional rate of mortality when concentration of infectious agents is [*V*]	Eqn 20
*μ*_*i*0_	Coefficient of [*V*] in additional mortality	0.02
*μ*_*i*1_	Coefficient of [*V*]^2^ in additional mortality	0.002
*r*_*v*_	Replication rate of infectious agents	0.05
*c*_*l*_	Clearance rate of infectious agents by lymphoid cells	0.05
*c*_*m*_	Clearance rate of infectious agents by myeloid cells	0
*v*_0_	Concentration of infectious agents at the start of an infection	1
*v*_*th*_	Concentration of infectious agents below which additional lymphoid mortality does not occur	0.025

(Parameter values are a canoncial fixed set, arbitrarily chosen, to illustrate the general principles of an MPCR).

Feedback control, which requires nonlinear dynamics, is essential for the growth and regeneration of tissues. A recent model of genetic products [21] to characterize the erythroid-myeloid lineage decision, shows how nonlinearities arise. de Graaf et al. [22] showed that the platelet concentration can regulate HSCs, through the concentration of Thrombopoietin (TPO). Other authors have shown that Lkb1, a kinase enzyme best known as a tumor suppressor, provides feedback control of HSCs [23-26]. Such work provides the empirical basis for our modeling.

To incorporate feedbacks we extend Lander et al.’s [27] approach: if *r* denotes a generic reaction rate constant and [*χ*] the concentration of fully differentiated cells, they model the reaction rate with feedback control is $\frac{r}{1+\nu [\chi ]}$. We have adapted this framework for the HSC system, with changes. First, two kinds of differentiated cells provide feedback; we use *l* and *m* to denote the total concentration of lymphoid and myeloid cells. Second, there is potentially different feedback on the activity of stem cells, the asymmetric differentiation of stem cells, and the activity of MPP cells. Third, the system needs to be active when there is a shortage of either lymphoid or myeloid cells. Thus we set:

(7) $$\begin{array}{ll} \Phi_{s}(l,m) & =\text{max}[\phi_{\text{sl}}(l),\phi_{\text{sm}}(m)]\; \end{array}$$

(8) $$\begin{array}{ll} \Phi_{p}(l,m) & =\text{max}[\phi_{\text{pl}}(l),\phi_{\text{pm}}(m)]\; \end{array}$$

(9) $$\begin{array}{ll} \Phi_{p^{\prime }}(l,m) & =\text{max}[\phi_{p^{\prime }l}(l),\phi_{p^{\prime }m}(m)]\; \end{array}$$

where *ϕ*_*sl*_(0) = *ϕ*_*sm*_(0) = 1(representing the feedback of lymphoid and myeloid cells on stem cell activity), etc, and all *ϕ*_*ij*_ are decreasing functions of their arguments, as in

(10) $$\begin{array}{ll} \phi_{\text{sl}}(l) & =\frac{1}{1+\alpha_{\text{sl}}\cdot l}\; \end{array}$$

(11) $$\begin{array}{ll} \phi_{\text{pl}}(l) & =\frac{1}{1+\alpha_{\text{pl}}\cdot l}\; \end{array}$$

(12) $$\begin{array}{ll} \phi_{p^{\prime }l}(l) & =\frac{1}{1+\alpha_{p^{\prime }l}\cdot l}\; \end{array}$$

where $\alpha_{\text{sl}},\alpha_{\text{pl}},\alpha_{p^{\prime }l}$

 are parameters. A similar form is used for the feedback control from myeloid cells. This is the simplest form of feedback between the whole organism and the bone marrow stem cell system; see [28] for alternatives.

In a demand control system the probability of a MPP cell ultimately differentiating into a CLP or CMP cell must depend upon the state of the organism. That is, the current densities of myeloid and lymphoid cells determine the appropriate response. We choose a functional form that is widely used in population biology and similar to Michaelis-Menten enzyme kinetics;

(13) $$\rho (l,m)=\frac{\alpha {\left(\frac{m}{l}\right)}^{\gamma }}{1+\alpha {\left(\frac{m}{l}\right)}^{\gamma }}$$

where *ρ*(*l*,*m*) represents the demand control function and *α*and *γ*are parameters that describe this specific asymptotic Michaelis-Menten function. We let *κ*_*m*_:*κ*_*l*_ denote the ratio of myeloid to lymphoid cells in homoeostasis. If *ρ*_*h*_denotes the value of *ρ*(*l*,*m*)in homeostasis then on average we have:

(14) $$\rho_{h}=\frac{\kappa_{l}}{\kappa_{l}+\kappa_{m}}$$

and from Eqn 13 we have

(15) $$\rho_{h}=\frac{\alpha {\left(\kappa_{m}/\kappa_{l}\right)}^{\gamma }}{1+\alpha {\left(\kappa_{m}/\kappa_{l}\right)}^{\gamma }}.$$

We view the unknowns in this equation as the two parameters *α* and *γ* from which we find

(16) $$\alpha =\left(\right)\frac{\rho_{h}}{1-\rho_{h}})){\left(\kappa_{m}/\kappa_{l}\right)}^{-\gamma }.$$

**Figure 2:**
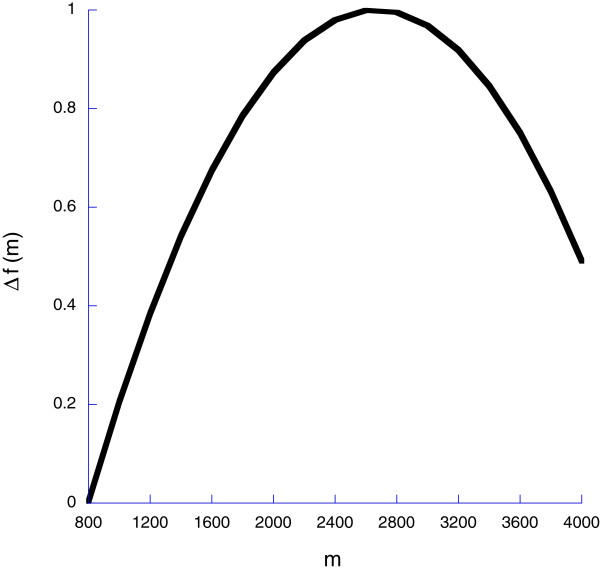
**a) The relationship between the parameters *α *and *γ *of the stem cell commitment response when homeostasis corresponds to 1 lymphoid cell per 1000 myeloid cells. b)** Different values of *γ*affect how the MPCR varies with changes in the number of lymphoid and myeloid cells. In the presence of high numbers of myeloid cells, the demand response is to drive the MPPs to make more lymphoid cells.

Eqn 16 determines a curve in the *γ*-*α*plane and every value on this curve provides the same value of *ρ*(*l*,*m*)=*ρ*_*h*_. However, when out of homeostasis, the value of *α*(*γ*)that determines Eqn 16 has a profound effect on the fate of HSC descendants. In Figure 2a we show the relationship between the parameters *α*and *γ* of the MPP commitment response when homeostasis corresponds to 1 lymphoid cell per 1000 myeloid cells, a typical ratio for humans. Each point on the lines in these panels correspond to a particular value of the pair (*γ*,*α*)consistent with the number of cells at homeostasis. In Figure 2b, we show how *ρ*(*l*,*m*) varies as *m*/*l* varies for three values of *γ*. As *γ*increases, the MPP commitment response becomes more sensitive to variation in the densities of myeloid and lymphoid cells. These three curves represent just a few possibilities in the infinite space of functional responses corresponding to Eqn 13 and raises the question: how can we predict which response an organism will use (i.e., where on the curve in Figure 2a will a population of organisms sit)? We discuss this below.

How an organism goes out of homeostasis depends upon its environment. For example, in an environment when wounds occur frequently, we anticipate the *m*/*l*will be lower than the value in homeostasis while in an environment when infection occurs frequently we anticipate that *m*/*l* will be greater than that value in homeostasis.

In this paper, we are interested in *ρ*(*l*,*m*), and in particular how natural selection affects it, in light of the environment of the organism. To do this, we need to couple Eqns 1-13 to organismal fitness, which is what separates our work from all that has come before it. We consider it advisable to step back from modeling a particular situation, but instead consider the more general properties that connect the needs of the organism with the activity of the bone marrow stem cell system.

### The components of fitness and their dynamics

**Figure 3:**
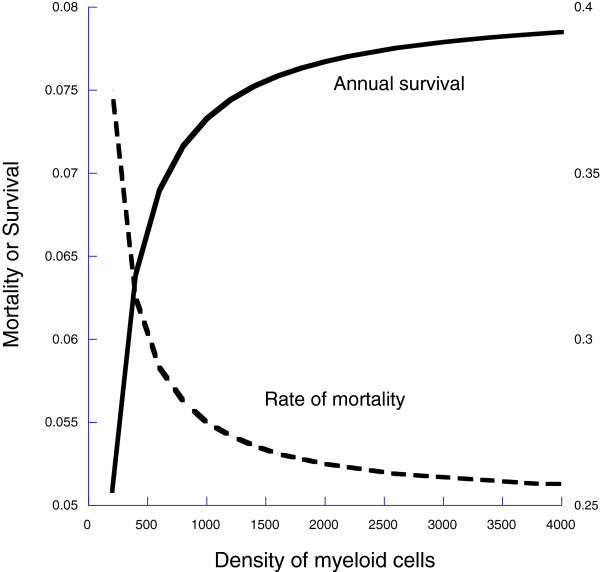
**We assume that the rate at which successful reproduction accumulates, *Δf *(*m*) is a parabolic function of the density of myeloid cells *m*.**

The representation of genes in subsequent generations is determined by survival and successful reproduction of the focal organism. Regarding the latter, we assume that the rate at which successful reproduction occurs (*Δf*([*M*])) is a function of myeloid cells, justified by the long recognition that organismal performance is a peaked function of hematocrit. Following figure two in [9], we set

(17) $$\mathrm{\Delta f}([M])=a_{0}+a_{1}\cdot M+a_{2}\cdot M^{2}$$

provided this expression is positive; otherwise we set *Δf*([*M*])=0, where *a*_0_=−1.034565, *a*_1_=0.001527, and *a*_2_=−0.0000002864. The peak of *Δf*([*M*]) occurs at [*M*]^∗^=2666(Figure 3). Hematocrit is the fraction of myeloid cells in blood; if too great then fitness is impaired as an organism has few lymphoid (immune) cells. If too low then there are insufficient myeloid cells to support the oxygen needs of the organism. Physiologically, the parabolic hematocrit function emerges through the relationship between the oxygen carrying capacity of blood and blood flow. When myeloid cells are low (*M*→0), blood flow is maximal but oxygen concentration is low. In contrast as *M*→*∞*, oxygen concentration is maximal but blood is too viscous to flow. Hence, this proximate physiological explanation and consequently the ultimate evolutionary fitnees cost lead to a peaked (parabolic) hematocrit function. The numerical relationship between reproductive rate and myeloid cell concentration described in equation (17) captures this parabolic shape.

We let F(t) denote lifetime fitness accumulated to time *t*, S(t) denote survival to time *t*, and *ε*<<1a scaling parameter that relates the organismal and cellular time scales. Then

(18) $$\frac{dF}{\text{dt}}=\varepsilon \cdot S(t)\cdot \mathrm{\Delta f}([M(t)])$$

**Figure 4:**
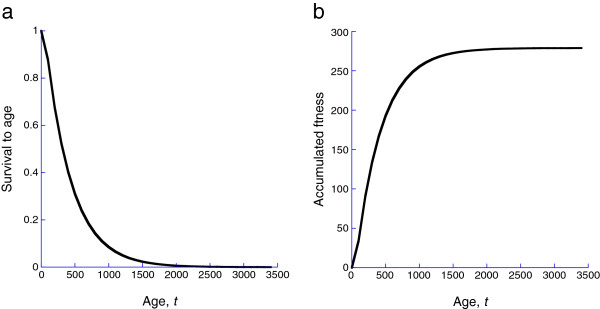
We assume that the rate of mortality declines with increasing numbers of myeloid cells, which has the effect that annual survival increases with increasing densities of myeloid cells; here we artificially hold the myeloid cells constant.

To determine survival, we assume that uninfected individuals have a per unit time rate of mortality with myeloid-independent and myeloid-dependent components so that the total rate of mortality is (Figure 4)

(19) $$\mu_{e}([M])=\mu_{e0}+\frac{\mu_{e1}}{\left[M\right]}$$

Although we focus on non-fatal diseases here, such diseases can still increase mortality rate, e.g. by reducing the effectiveness of flight responses. Hence we assume that the additional mortality induced by the pathogen is

(20) $$\mu_{i}([V])=\mu_{i0}[V]+\mu_{i1}{\left[V\right]}^{2}$$

so that

(21) $$\frac{dS}{\text{dt}}=-\varepsilon \cdot (\mu_{e}([M])+\mu_{i}[V])S$$

Finally, we incorporate the dynamics of infectious agents. We assume that in the absence of immune response the growth of the infectious agent is exponential with rate *r*_*v*_ and that lymphoid and myeloid cells clear the infection at rate *c*_*l*_and *c*_*m*_respectively. In this model, again for simplicity, we ignore memory in the immune system. Thus, if *V*(*t*) denotes the density of pathogens

(22) $$\frac{\text{dV}}{\text{dt}}=\;[r_{v}-c_{l}L(t)-c_{m}M(t)]\cdot V(t)$$

Integration of Eqns 17-22 forward in time, conditioned on *ρ*([*L*],[*M*]) and a sequence of wound and/or infection events, allows us to compute the lifetime reproductive success associated with that particular MPP commitment response.

Eqns 1-22 are a set of deterministic ordinary differential equations that link the behavior of the stem cell system with the needs of the organism. However, organisms in nature experience wounding and infection in a quasi-random manner. We account for this in the following way. Imagine that there are *K*_*w*_ and *K*_*i*_times at which wounds or infections can occur (these values could, of course, be random variables but we treat them as fixed in this paper, only for purposes of simplicity) and then determine a sequence of times *T*_*w*_(*k*_*w*_),*k*_*w*_=1,2,*....*,*K*_*w*_ and *T*_*i*_(*k*_*i*_),*k*_*i*_=1,2,*....*,*K*_*i*_ at which either a wound or infection occurs (in principle both could occur at one time). To illustrate the ideas, we assume that when a wound occurs, myeloid cells drop by 40% and that when an infection occurs, the infectious agent increases to the level *v*_0_. These occur instantaneously and we then continue with the solution of the differential equations. For the results shown here, we assume that *K*_*w*_=*K*_*i*_=7 and that the times are uniformly distributed over the interval between day 0 and day 1500.

## Results

As introduced above (Figure 2a) a homeostatic *ρ*_*h*_ratio of fully differentiated myeloid to lymphoid cells specifies a curve in the *γ*−*α* plane in which all points on this curve are consistent with *ρ*_*h*_ but as illustrated in Figure 2b, different values of *γ*(and thus *α*) will lead to different MPP commitment responses when the organism is out of homeostasis. Thus, to understand how natural selection will act on *ρ*(*l*,*m*)requires dealing explicitly with components of fitness and linking them to the dynamics of the HSC system.

To explore this hypothesis, we assume that the rate at which the organism accumulates fitness, *Δf*(*m*) is a parabolic function of myeloid cells (Figure 3) and that the rate of mortality is a declining function of myeloid cells (Figure 4) and an increasing function of the density of infectious agents (Eqn 22).

**Figure 5:**
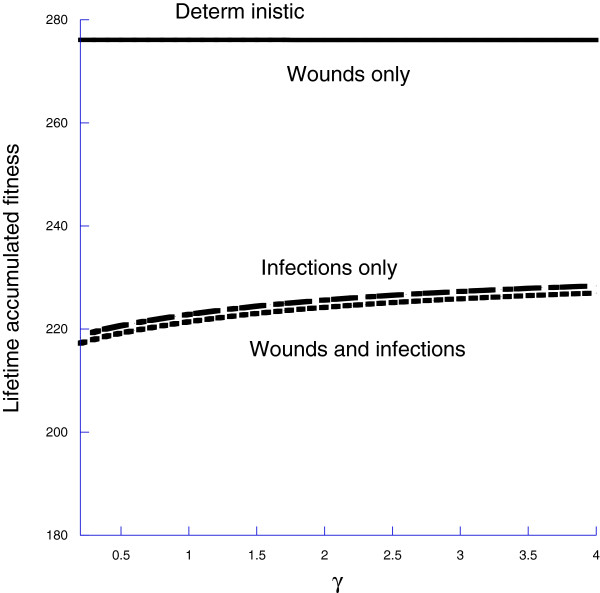
Even in a laboratory environment, without wounding or infection, organism do not live forever, so that survival declines with age (panel a) with the consequence that accumulated fitness saturates.

**Figure 6:**
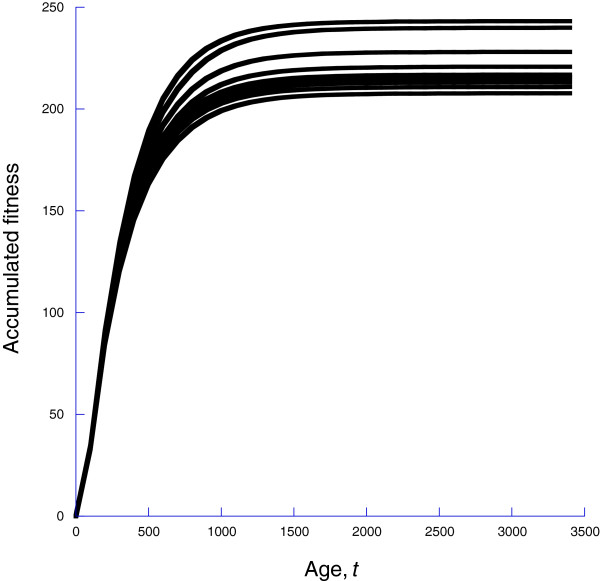
**Plotting lifetime accumulated fitness as a function of *γ *allows us to understand the strength of selection on *γ*as determined by the environment in which the organism lives.**

In a ‘deterministic’ or laboratory environment with neither wounding nor infection, organisms still die, so that survival declines with age (Figure 5a) and fitness accumulates but ultimately saturates because of the declining survival (Figure 5b). The strength of selection on *ρ*(*l*,*m*) will depend upon how fitness varies with *γ*. We show this in Figure 6 fitness as a function of *γ*for the deterministic case, wounding only, infections only, and wounding and infections. Clearly there is little selection on *γ* in the laboratory case or of wounding only. Selection does occur when there are infections, with larger values of *γ*, leading to a more responsive MPCR.

**Figure 7:**
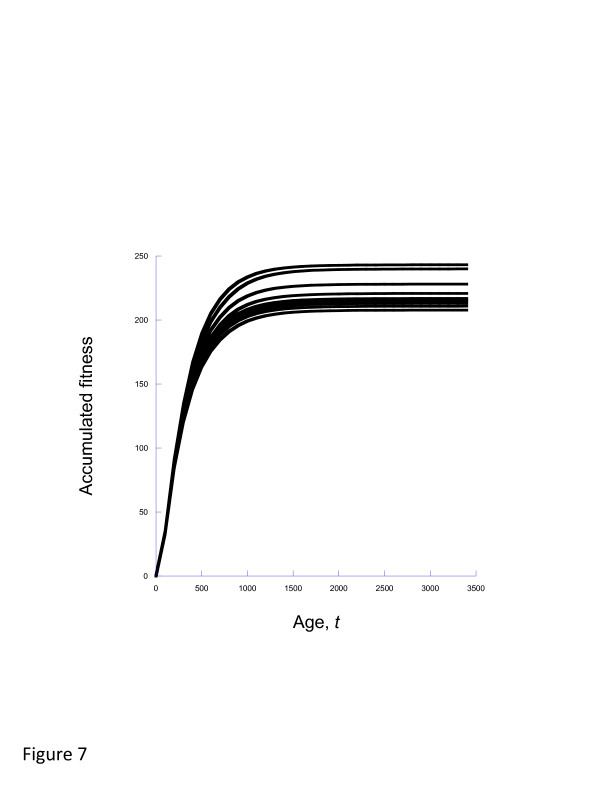
**Ten realizations of the model with both wounds and infection, for the case of *γ *= 2.**

The alternative to varying *γ*and holding the environment at one stochastic realization is to hold *γ* constant and consider multiple realizations of the stochastic environment. We show the results of such an approach in Figure 7, for *γ*=2. Using these, we can compute both a mean and variance for the selection acting on *γ*.

## Discussion

Here we have developed and analysed a theoretical framework for linking the population biology of the hematopoietic stem cells to the demands of the individual. We have introduced the notion of the MPCR (Multipotent Progenitor Commitment Response) as the response that the describes the penultimate decision of stem cells before commitment to either a myeloid or lymphoid lineage. We use this response to investigate the control dynamics of a hematopoietic stem cell system and show that different values of the ‘shape’ parameters that describe the MPCR give a range of optimal response. Below we discuss the implications of this on the evolutionary dynamics of the HSC system and, more broadly, for developing a theory of stem cell systems based in population biology.

### The meaning of a flat fitness surface

The first derivative, $\frac{\partial F}{\partial \gamma }$, of the accumulated fitness with respect to *γ*is a fundamental measure of the strength of natural selection on *γ*. As the derivative becomes smaller, the strength of natural selection becomes weaker, with the implication that a wider spectrum of values of *γ*will provide equal values of fitness. But, as we have shown in Figure 2b, differing values of *γ* will lead to considerably different MPCRs, and thus kinetics of the HSC descendants following an external challenge such as a transplant or perturbation, with the prediction that if one uses animals with little evolutionary history of wounding or infection, a wide range of HSC dynamical responses is expected. For instance, among 44 laboratory mice, Abkowitz et al. [29] observed seven different patterns of donor cell dynamics following hematopoeitic stem cell transplant experiments, suggesting that there is individual heterogeneity in the parameters of the MPCR, as we would predict.

In previous work [15] we showed that the differential equations used here are a good approximation for the mean of underlying stochastic system. Understanding the limitations imposed by stochastic fluctuations on the feedback in our model [30] is an important next step because the comparisons of models and data will require a framework to account for process stochasticity and observation error. Developing appropriate state-space approaches to understand the consequences of these heterogeneities and nonlinearities on stem cell dynamics is clear and obvious future step.

Recently, Huang [31] proposed that a systems biology of stem cells should consider not only the link between observation and process through state-space approaches (e.g. [32]) but also the consequences of nonlinearity and non-genetic heterogeneity in cell systems. Here, to start to understand how nonlinearity effects influence stem cell behavior we have introduced the notion of a MPCR under the control from the needs of the organism. Furthermore, under a paradigm of stem cell heterogeneity, we should anticipate variation at multiple levels within any stem cell system; particularly so with individual variation in the MPCR (*γ* and *α*).

### How this model can be extended

There are a number of extensions that go beyond the current work for linking population biology and stem cell systems. For instance, experimental validation of the MPCR would require repeated cell count measures of long-term HSCs, short-term HSCs and a range of HSC-derived products. Given such experimental data we envisage that it would be possible to assess goodness of fit between an MPCR model and data (and also characterize unexplained heterogeneties) using computational and statistical methods (Bonsall and Mangel, unpublished).

Furthermore, our framework could be extended to study the consequences of transplant or perturbation effects A perturbation experiment can be modeled by starting the HSC system in its steady state and then reducing the number of lymphoid or myeloid cells and then integrating Eqns 1-22 forward. In addition to predicting the kinetics of fully differentiated cells, we can predict the activity of the stem cells following the perturbation. As described in the Additional file 1, a transplant experiment involves the addition of the dynamics of host cells, which are dying, and allows us to predict the fraction of fully developed lymphoid or myeloid cells. Our approach can also be used, in conjunction with evolutionary invasion analysis as in [15], to predict the outcome of a partial transplant of stem cells, in which stem cells with different kinetic properties are transplanted into an organism with a healthy but not vibrant stem cell system and for which we wish to predict whether the transplanted stem cells will co-exist with the host stem cells, overtake them, or be extinguished by them.

### Computing *ρ*(*l*,*m*)from more fundamental principles: the fitness control hypothesis

We began with the assumption that $\rho (l,m)=\frac{\alpha {\left(\frac{m}{l}\right)}^{\gamma }}{1+\alpha {\left(\frac{m}{l}\right)}^{\gamma }}$, but there are an infinite number of functions that would have the properties we seek: bounded by 1 and increasing as the the ratio *m*/*l*increases. Given experimental data, it is possible to use Bayesian methods to determine *ρ*(*l*,*m*) (Bonsall and Mangel, unpublished), but we may also ask if *ρ*(*l*,*m*)could be constructed from some more fundamental principles. One way to do approach this woul be through state dependent life history, as implemented by stochastic dynamic programming (SDP) [17-20], which we explain now but leave the details for a subsequent paper.

Our results point to organismal fitness being a function of fully differentiated lymphoid and myeloid cells, F(l,m,t), which we have modeled (Eqns 17-22). Beginning there, consider the question from the perspective of the organism, rather than from the perspective of the dynamics of cells. Suppose that *s* denotes the time scale for the organism, so that in the interval *s* to *s* + 1 the time *Δt*>>1elapses in the cellular model. During that elapsed time, we assume that up to P fully differentiated cells are produced and the challenge for the organism is to allocate these fully differentiated cells to lymphoid or myeloid cells. That is, if *δl* and *δm* denote the number of fully differentiated cells produced during *Δt*, subject to δl+δm≤P, then we seek the value of P and combination of *δ*_*l*_and *δ*_*m*_ that maximizes organismal fitness.

To do this, we let *F*(*l*,*m*,*s*)denote the accumulated lifetime reproduction from time *s* until time *S* given that [*L*(*s*)]=*l*,[*M*(*s*)]=*m* and the organism is not infected. Similarly, we let *F*_*i*_(*l*,*m*,*v*,*s*)denote accumulated lifetime reproduction under the same conditions about lymphoid and myeloid cells and that the density of infectious agents is [*V*(*s*)]=*v*. Since fitness cannot be accumulated after time *S*, we have the end condition *F*(*l*,*m*,*S*)=*F*_*i*_(*l*,*m*,*v*,*S*)=0. Methods of SDP, along with value iteration, allow us to compute the stationary values of these functions for *s*<<*S*[15].

Such a method allows us to represent the optimal production of CLP and CMP cells given that the organism is not infected at time *s* and that [*L*(*s*)]=*l*and *M*[*s*]=*m*. Then

(23) $$P_{\prime }^{*}(l,m,s)=\delta_{0}l^{*}(l,m,s)+\delta_{0}m^{*}(l,m,s)$$

is the optimal production of MPP cells at time *s*, given the current state of the organism. Similar arguments generate the optimal production of MPP cells, distributed as CLP or CMP for the case of the organism being infected at time *s*.

In the stationary state, fitness is only a function of *l*,*m*and *v*; we denote these values by $\bar{F}(l,m)$ and ${\bar{F}}_{i}(l,m)$. Similarly, the analog of the quantities in Eqn 23 are ${\bar{\delta }}_{0}l^{*}(l,m)$, ${\bar{\delta }}_{0}m^{*}(l,m)$ and ${\bar{P}}^{*}(l,m)$. Thus, for an organism that is currently not infected, the fraction of CLP cells produced is

(24) $$\rho (l,m)=\frac{{\bar{\delta }}_{0}l^{*}(l,m)}{{\bar{\delta }}_{0}l^{*}(l,m)+{\bar{\delta }}_{0}m^{*}(l,m)}$$

This equation provides an explicit form for *ρ*(*l*,*m*)based directly on fitness of the organism, with a similar one holding for the situation in which the organism is infected.

### Connection to empirical studies

Our work complements the rapid recent development of understanding how gene products that regulate HSCs operate [33]. However, translating that understanding into a functional perspective at the organismal level is less well developed. Recent work suggests that epigenetic factors [34] are important in controlling HSC behavior [35], but the scale of those factors is unknown, and evidence suggests that HSC behavior is regulated by circadian oscillations modulated by photic cues [36], as is the behavior of whole organisms [37]. Other the hand, it is less clear how the differentiated T and B cells are regulated in adaptive immunity [38]. DNA damage increases in HSCs with age and such damage is more poorly repaired in older individuals [39,40]; lymphocytes also age [41]. Rossi et al. [42] argue that the reduction in adaptive immune capacity with age and the increased incidence of myeloproliferative diseases both have origins in changes in the HSC system, presumably due to increased damage of HSCs that could include telomere shortening or other genome maintenance failures, and other damage to DNA or proteins [43-46].

Thus, currently much is known about the mechanism of HSC system and its descendants [47,48] but much less is known about how natural selection has shaped these mechanisms, even though stem cells are units of natural selection [49]. Using classic ecological methods of allometric analysis (e.g., [50]) and compartmental modeling, it has recently been argued [51-53] that the fundamental architecture of the HSC system is unchanged across mammalian species (also see [54]), which suggests a long evolutionary history determining the role of HSCs in organismal ecology. Recognizing that evolutionary considerations are essential for understanding every problem in biology [55], our goal here has been to understand how the penultimate route of differentiation towards a common myeloid or lymphoid progenitor cell [56-58] can be characterized both dynamically and evolutionarily.

One common way for approaching an understanding of the dynamics of HSCs and their products is through irradiation and transplant experiments (e.g., [59]), which also have important implications for bone marrow transplants [60,61]. The typical experiment proceeds as follows [61]: a host animal receives a dose of irradiation sufficient to destroy the blood system (about 900 rad) but which leaves the rest of the organ systems intact (the intestinal system requires 1200-12,000 rad for irreversible damage, and the brain more than 12,000). This dose will kill the animal within 2-3 weeks, which gives a sense of the rate of decline of HSC descendants. Cells of a donor are isolated from the bone marrow by flushing sterile medium through the bone marrow and then injected into the host animal. The donor cells first circulate in the blood stream and then repopulate the bone marrow. The success of such transplants, from the perspective of the whole organism is demonstrated when a skin graft from the donor to the host is not rejected. In the period following transplantation, the donor HSCs produce progenitors that then differentiate into myeloid or lymphoid cells. Each differentiation that leads to a commitment of one route or another involves complex signaling [56,57,62,63] and the signals themselves must be shaped by natural selection. We call this a transplant experiment.

A less commonly used approach, but equally important, is a perturbation experiment in which an animal is challenged in a way that reduces its complement of erythrocytes, platelets or granulocytes (commom myeloid progenitor (CMP) descendants) or lymphocytes (common lymphoid progenitor (CLP) descendants) and HSC activity is observed subsequent to the perturbation. For example, Cheshier et al. [10] bled mice on days 3, 6, and 9 and then sacrificed them on day 10 and measured the markers of HSC activity (see [64] for a similar experiment regarding the epidermis). Baldridge et al. [11] showed that quiescent HSCs were activated in response to an infection, another perturbation experiment. The models that we have described here can be used to predict dynamics of stem cells and their descendants for both kinds of experiments.

## Conclusions

The use of quantitative models to understand the HSC system can be traced to the classic work of Till et al. [65], who used branching processes to interpret their experimental results on the variation of spleen colonies formed after a transplant experiment but evolutionary considerations rarely appear in models in stem cell biology [15]. The concepts developed for understanding how populations of individuals respond to these abiotic and biotic processes have colloraries at the cellular level. In particular, the size of stem cell systems is determined by the availability of molecular resources, feedbacks from differentiated cells, the effects of the epigenetic environment and the size of the environment capable of supporting stem cells [66]. Finally, population biology forces us to recognize the distinction between typological thinking (that all individuals of the same species are identical with constant characteristics and responses) and population thinking (that variation is real because every individual is unique and individual variation is central in ontogenetic and evolutionary history) [67]. While there are verbal and pictorial models (e.g.,[5,68,69]) of how signaling between differentiated cells and HSC may work, we have provided the first general quantitative theory linking how feedbacks through organismal need shapes the performance of the HSC system (cf [70]).

In conclusion, our work raises the critical question of how we connect the MPCR with the vast understanding on how signalling shapes HSC products. For instance, appreciating how the MPCR links to T-cell specification [71] and drives differential levels of the several well characterised precursors (of which lymphoid primed MPPs are one) requires further consideration of the both the key qualitative and quantitative positive and negative feedbacks in these sorts of signalling pathways. Similarly, linking the ideas of demand feedback to erythropoietin production [72], that differs between foetus and adult, requires systematic approach to combining the dynamical drivers with the lifetime fitness of the organism.

While the broad evolutionary ecological of HSC activity such as mounting an immune response to infection are well-known to have a cost on reproductive fitness (e.g., [73]), appreciating the ecological setting suggests that standard laboratory animals (bred for many generations under refined conditions) may not be the most appropriate organisms for the sorts of empirical tests of the theory we propose (also see [74] on metabolic morbidity of laboratory rodents). Studies on the evolutionary ecology of model organisms (see [75] for an approach using zebrafish) should have greater prominence in the understanding the population biology of stem cell responses.

Although raised almost two decades ago [76] this systems biology approach to stem cell dynamics must include evolutionary thinking. Approaching stem cell biology as population biology has much to offer both fields.

## Competing interests

The authors have declared that no competing interests exist.

## Authors’ contributions

The model was conceived and designed jointly (MM, MBB). MM analyzed the model and developed the results. The paper was wrote jointly (MM, MBB). Both authors read and approved the final manuscript.

## Supplementary Material

Additional file 1 Details of the HSC model derivation. Click here for file
