# Supplementary material for: Stem cell biology is population biology: differentiation of hematopoietic multipotent progenitors to common lymphoid and myeloid progenitors
Source: Theor Biol Med Model. 2013 Jan 17;10:5. doi: 10.1186/1742-4682-10-5 (PMC3765094; doi:10.1186/1742-4682-10-5)
Supplement: Additional file 1 — Details of the HSC model derivation. [file 1742-4682-10-5-S1.pdf]

Stem cell biology is population biology: differentiation of hematopoietic multipotent progenitors to common lymphoid and myeloid progenitors

Marc Mangel[1] and Michael B. Bonsall [2]

December 21, 2012

1. Department of Applied Mathematics and Statistics, University of California, Santa Cruz, CA and Department of Biology, University of Bergen, Bergen, Norway. Email: msmangel@ucsc.edu. Corresponding author a

2. Mathematical Ecology Research Group, Department of Zoology, University of Oxford and St Peter's College, Oxford, UK, Email: michael.bonsall@zoo.ox.ac.uk

Additional File 1 – Supplementary Material

In this supplement, we show how the full set of cellular dynamics shown in Figure 1A in the main text can be simplified to Eqns 1-6 in the main text. Following [77-80], we describe cell dynamics using reaction kinetics, from which we derive ordinary differential equations that characterize the population dynamics of cell numbers. In [15], we gave a simplified stochastic version of the reaction kinetics that follow; we use a deterministic framework here because of the additional complexities we investigate. We first give the equations without feedback control and then explain the nature of the feedback control.

Stem Cells and Multipotent Progenitor Cells

We let S and MPP_j denote stem cell and the jth ($j = 0, 1, 2, ...N$) state of multipotent progenitor cells.

In the absence of feedback control, the reactions characterizing the dynamics of these cells are

$$S \xrightarrow{r_s} 2S \tag{1}$$

$$S \xrightarrow{r_p} S + MPP_0 \tag{2}$$

$$S \xrightarrow{r_{p'}} 2 \cdot MPP_0 \tag{3}$$

$$MPP_j \xrightarrow{r_j} MPP_{j+1} \tag{4}$$

$$MPP_j \xrightarrow{\lambda_j} 2 \cdot MPP_j \tag{5}$$

We assume that MPP proliferation declines as the terminal differentiation is approached, which means that λ_j declines as j increases.

Common Lymphoid Progenitors and Common Myleoid Progenitors

After the Nth intermediate multipotent progenitor state, a MPP cell differentiates into a Common Lymphoid Progenitor (CLP) or Common Myeloid Progenitor (CMP) cell. Suppressing the dependence upon the concentrations of fully differentiated lymphoid and myeloid cells, we let $0 \leq \rho \leq 1$ denote the fraction of MPP differentiations that follow the CLP route then the reaction kinetics are

$$MPP_N \xrightarrow{\rho r_{d,MPP}} CLP \tag{6}$$

$$MPP_N \xrightarrow{(1-\rho) r_{d,MPP}} CMP \tag{7}$$

Fully Differentiated Cells

We consider the following simplified system of fully differentiated cells: 1) fully differentiated lymphoid cells are B cells (B), T cells (T), and natural killer cells (NK); 2) fully differentiated megakaryocytes are:erythrocytes (E) and platelets (P); and 3) fully differentiated granulocytes (G) combine neutrophils, esoinophils, basophils, mast cells, and macrophages. It is clear how to remove the simplifications by expanding the granulocyte class if one wishes complexity before simplicity.

With obvious interpretation of the new parameters, we write

$$CLP \xrightarrow{\rho_B r_{CLP}} B \tag{8}$$

$$CLP \xrightarrow{\rho_{NK} r_{CLP}} NK \tag{9}$$

$$CLP \xrightarrow{(1-\rho_B-\rho_{NK}) r_{CLP}} T \tag{10}$$

$$CMP \xrightarrow{\rho_E r_{CMP}} E \tag{11}$$

$$CMP \xrightarrow{\rho_G r_{CMP}} G \tag{12}$$

$$CMP \xrightarrow{(1-\rho_E-\rho_G) r_{CMP}} P \tag{13}$$

The total number of fully differentiated lymphoid cells, denoted by L, is the sum of the number of B, T, and NK cells and the total number of myeloid cells, denoted by M is the sumer of the number of E, P, and G cells. In evolutionary ecology, such sums would be called trophic species, since although there are differences in the cell (species) populations, they play the same general role in the ecosystem (organism).

Feedback Control

We do not explicitly model the cytokine-based feedback between the fully differentiated cells and the stem cells, nor the feedback within the niche. Instead, we follow [27] and modify the reaction rates, so that instead of being constant, they depend on the levels of fully differentiated products.

We assume that the niche can support at most K stem cells and that in absence of all other feedback, the dynamics in the niche follow Gompertzian kinetics (justified in [15]). In addition to the within-niche feedback control, we let $\Phi_s(L,M)$ denote the feedback control from the fully differentiated cells [using the short-hand L, M to avoid writing 6 arguments], so that r_s in Eqn 1 is replaced by $r_s \cdot S \cdot log(K/s) \cdot \Phi_s(L,M)$. Similarly r_p in Eqn 2 is replaced by $r_p \cdot \Phi_s(L,M)$. We assume that there is additional feedback control on asymmetric differentiation of stem cells (Eqn 3) so that $r_{p'}$ is replaced by $r_{p'}\Phi_s(L,M)\Phi_{p'}(L,M)$. We assume that feedback control acts on each stage of MPP development so that each of the λ_j, r_j are replaced by $\lambda_j\Phi_p(L,M)$. and $r_j\Phi_p(L,M)$.

We will describe the specific form of the feedback functions after giving the fully general reaction rate equations.

The Reaction Rate Equations

We let μ_i denote the rate of mortality of cell type i (if fully developed cells also proliferate, we can capture that by setting $\mu_i < 0$) and [ ] concentration of cells. The dynamics of the full system is then

$$
\begin{aligned}
\frac{d[S]}{dt} &= [S] \cdot log(K/[S])(r_s - r_{p'}\Phi_{p'}([L],[M]))\Phi_s([L],[M]) - \mu_s[S] && (14)\\
\frac{d[MPP_0]}{dt} &= [S] \cdot log(K/[S])(r_p + 2r_{p'}\Phi_{p'}([L],[M]))\Phi_s([L],[M]) && (15)\\
&+(\lambda_0 - r_0)\Phi_p([L],[M])[MPP_0] - \mu_0[MPP_0] &&\\
\frac{d[MPP_j]}{dt} &= r_{j-1}\Phi_p(L,M)[MPP_{j-1}] && (16)\\
&+(\lambda_j - r_j)\Phi_p(L,M)[MPP_j] - \mu_j[MPP_j], j = 1,2,..N-1 &&\\
\frac{d[MPP_N]}{dt} &= r_{N-1}\Phi_p(L,M)[MPP_{N-1}] && (17)\\
&+(\lambda_N - r_{d,MPP})\Phi_p(L,M)[MPP_N] - \mu_N[MPP_N] &&\\
\frac{d[CLP]}{dt} &= r_{d,MPP}\Phi_p(L,M)\rho([L],[M])[MPP_N] - r_{CLP}[CLP] - \mu_{CLP}[CLP] && (18)\\
\frac{d[CMP]}{dt} &= r_{d,MPP}\Phi_p(L,M)(1-\rho([L],[M]))[MPP_N] && (19)\\
&-r_{CMP}[CMP] - \mu_{CMP}[CMP] &&\\
\frac{d[B]}{dt} &= r_{CLP}\rho_B([B],[NK],[T])[CLP] - \mu_B[B] && (20)\\
\frac{d[NK]}{dt} &= r_{CLP}\rho_{NK}([B],[NK],[T])[CLP] - \mu_{NK}[NK] && (21)\\
\frac{d[T]}{dt} &= r_{CLP}(1-\rho_B([B],[NK],[T]) - \rho_{NK}([B],[NK],[T]))[CLP] - \mu_T[T] && (22)\\
\frac{d[E]}{dt} &= r_{CMP}\rho_E([E],[G],[P])[CMP] - \mu_E[E] && (23)\\
\frac{d[G]}{dt} &= r_{CMP}\rho_G([E],[G],[P])[CMP] - \mu_G[G] && (24)\\
\frac{d[P]}{dt} &= r_{CMP}(1-\rho_E([E],[G],[P]) - \rho_G([E],[G],[P]))[CMP] - \mu_P[P] && (25)
\end{aligned}
$$

Rapid Development and Differentiation of Progenitor Cells and Lumped Myeloid and Lymphoid Cells

If the development rate of MPP cells is fast (either because the reactions are fast or N is small), we can use a quasi-steady state analysis as is commonly done for Michaelis-Menten kinetics [81]. We then write

Eqn 16 as

$$\frac{1}{r_j}\frac{d[MPP_j]}{dt} = \Phi_p(L,M)[MPP_{j-1}] + [(\frac{\lambda_j}{r_j} - 1)\Phi_p(L,M) - \frac{\mu_j}{r_j}][MPP_j] \tag{26}$$

If we assume that the left-hand side of Eqn 26 and $\frac{\mu_j}{r_j}$ are much less than 1, then Eqn 26 simplifies to

$$[MPP_j] = \frac{r_{j-1}}{r_j}(1 - \frac{\lambda_j}{r_j})^{-1}[MPP_{j-1}] \tag{27}$$

Applying a similar analysis to Eqn 17 leads to

$$[MPP_N] = \frac{r_{N-1}}{r_d} \cdot (1 - \frac{\lambda_N}{r_d})^{-1} \prod_{j=1}^{N-1} \frac{r_{j-1}}{r_j}(1 - \frac{\lambda_j}{r_j})^{-1}[MPP_0] \tag{28}$$

which we write as

$$[MPP_N] = \Omega_N[MPP_0] \tag{29}$$

with the obvious definition of Ω_N. Eqns 18 and 19 become

$$\frac{d[CLP]}{dt} = r_{d,MPP}\Phi_p(L,M)\rho([L],[M])\Omega_N[MPP_0] - r_{CLP}[CLP] - \mu_{CLP}[CLP] \tag{30}$$

$$\frac{d[CMP]}{dt} = r_{d,MPP}\Phi_p(L,M)(1 - \rho([L],[M]))\Omega_N[MPP_0] \tag{31}$$

$$-r_{CMP}[CMP] - \mu_{CMP}[CMP]$$

$$\tag{32}$$

We now denote progenitors by MPP, lump B, NK, and T cells into a lymphoid class, with concentration denoted by $[L]$ and E, G, and P cells into a myeloid class, with concentration denoted by $[M]$, equations 14-25 simplify to

$$
\begin{aligned}
\frac{d[S]}{dt} &= [S]\cdot log(K/[S])(r_s - r_{p'}\Phi_{p'}([L],[M]))\Phi_s([L],[M]) - \mu_s[S] &\quad (33)\\
\frac{d[MPP]}{dt} &= [S]\cdot log(K/[S])(r_p + 2r_{p'}\Phi_{p'}([L],[M]))\Phi_s([L],[M]) &\quad (34)\\
&\quad +(\lambda - r_{d,MPP})\Phi_p([L],[M])[MPP] - \mu_p[MPP] \\
\frac{d[CLP]}{dt} &= r_{d,MPP}\Phi_p(L,M)\rho([L],[M])\Omega_N[MPP] &\quad (35)\\
&\quad -r_{CLP}[CLP] - \mu_{CLP}[CLP] \\
\frac{d[CMP]}{dt} &= r_{d,MPP}\Phi_p(L,M)(1-\rho([L],[M])])\Omega_N[MPP] &\quad (36)\\
&\quad -r_{CMP}[CMP] - \mu_{CMP}[CMP] \\
\frac{d[L]}{dt} &= r_{CLP}[CLP] + (r_l - \mu_l)[L] &\quad (37)\\
\frac{d[M]}{dt} &= r_{CMP}[CMP] + (r_m - \mu_m)[M]. &\quad (38)
\end{aligned}
$$

It is these equations that form the bases of the analysis in the main text, and complement the variety of other models of the dynamics of the HSC system (Supplementary Table 1)

Supplementary Table S1 An Overview of Some Key Models Applied to HSCs and Their Descendants

Source	Kind of Model	Focus
(65) Till et al 1964	Branching Process	Origins of Variability
(82) Vogel et al 1969	Stochastic branching processes	Development of erythroblastic colonies
(83) Mackey 1978	Nonlinear ordinary differential equations for cycling cells	Aplastic anemia
(76) Novak and Necas 1994	Steady state algebraic analysis	Lineage proliferation in the steady state
(84) Abkowitz et al 1996	Markov Birth and Death Process	Evidence that hematopoiesis is stochastic
(85) Haurie et al 1998	Delay differential equations	Origins of periodic hematological disorders
(29) Abkowtiz et al 2000	Stochastic comparent model	Patterns of individual variation following transplants
(86) Bernard et al 2004	Nonlinear ordinary differential equations	Origins of oscillatory WBC diseases
(87) Colijn and Mackey 2005	Delay-differential equations	Oscillating leukemia
(52) Dingli and Pacheco 2006	Allometric scaling methods	Estimating the size of the active HSC pool
